# Routine health management information system data in Ethiopia: consistency, trends, and challenges

**DOI:** 10.1080/16549716.2020.1868961

**Published:** 2021-01-15

**Authors:** Abyot Adane, Tewabe M. Adege, Mesoud M. Ahmed, Habtamu A. Anteneh, Emiamrew S. Ayalew, Della Berhanu, Netsanet Berhanu, Misrak G. Beyene, Antoinette Bhattacharya, Tesfahun Bishaw, Eshetu Cherinet, Mamo Dereje, Tsega H. Desta, Abera Dibabe, Heven S. Firew, Freweini Gebrehiwot, Etenesh Gebreyohannes, Zenebech Gella, Addis Girma, Zuriash Halefom, Sorsa F. Jama, Binyam Kemal, Abyi Kiflom, Carina Källestål, Seblewengel Lemma, Yidnekachew D. Mazengiya, Kalkidan Mekete, Magdelawit Mengesha, Meresha W. Nega, Israel A. Otoro, Joanna Schellenberg, Tefera Taddele, Gulilat Tefera, Admasu Teketel, Miraf Tesfaye, Tsion Tsegaye, Kidist Woldesenbet, Yakob Wondarad, Zemzem M. Yosuf, Kidist Zealiyas, Mebratom H. Zeweli, Lars Åke Persson, Annika Janson

**Affiliations:** a Ethiopian Pharmaceutical Supply Agency, Addis Ababa, Ethiopia; b Ministry of Health, Addis Ababa, Ethiopia; c Department of Disease Control, London School of Hygiene & Tropical Medicine, London, UK; d Ethiopian Public Health Institute, Addis Ababa, Ethiopia; e Department of Women’s and Children’s Health, Uppsala University, Uppsala, Sweden; f Department of Women´s and Children’s Health, Karolinska Institutet, Stockholm, Sweden

**Keywords:** Data Quality, HMIS, RHIS, Routine Health Information System, WHO data quality review toolkit

## Abstract

**Background**: Ethiopia is investing in the routine Health Management Information System. Improved routine data are needed for decision-making in the health sector.

**Objective**: To analyse the quality of the routine Health Management Information System data and triangulate with other sources, such as the Demographic and Health Surveys.

**Methods**: We analysed national Health Management Information System data on 19 indicators of maternal health, neonatal survival, immunization, child nutrition, malaria, and tuberculosis over the 2012–2018 time period. The analyses were conducted by 38 analysts from the Ministry of Health, Ethiopia, and two government agencies who participated in the Operational Research and Coaching for Analysts (ORCA) project between June 2018 and June 2020. Using a World Health Organization Data Quality Review toolkit, we assessed indicator definitions, completeness, internal consistency over time and between related indicators, and external consistency compared with other data sources.

**Results**: Several services reported coverage of above 100%. For many indicators, denominators were based on poor-quality population data estimates. Data on individual vaccinations had relatively good internal consistency. In contrast, there was low external consistency for data on fully vaccinated children, with the routine Health Management Information System showing 89% coverage but the Demographic and Health Survey estimate at 39%. Maternal health indicators displayed increasing coverage over time. Indicators on child nutrition, malaria, and tuberculosis were less consistent. Data on neonatal mortality were incomplete and operationalised as mortality on day 0–6. Our comparisons with survey and population projections indicated that one in eight early neonatal deaths were reported in the routine Health Management Information System. Data quality varied between regions.

**Conclusions**: The quality of routine data gathered in the health system needs further attention. We suggest regular triangulation with data from other sources. We recommend addressing the denominator issues, reducing the complexity of indicators, and aligning indicators to international definitions.

## Background

A routine Health Management Information System (HMIS) ideally provides accurate, disaggregated, and real-time information from all health system levels to enable disease surveillance, activity monitoring, allocation of resources, and policy formation. It can also inform patients and provide feedback to professionals in the health-care system. In the absence of a well-functioning routine HMIS, most low- and middle-income countries rely upon survey data, such as the Ethiopian Demographic and Health Surveys (EDHS) [1,2]. Such studies usually produce valid and reliable information. However, these surveys are costly, retrospective, and intermittent, which makes their results less suitable for guiding current planning and policy formation [3]. In most cases, national surveys do not provide district-level data for health planning.

The Information Revolution was one of the four transformation agendas in Ethiopia’s first Health Sector Transformation Plan [4,5]. The information revolution aimed to advance collection, analysis, presentation, and dissemination of information that could influence decision-making. A particular focus was given to the introduction of new information technology, including the computer software District Health Information System (DHIS2), used in over 60 countries [6,7]. However, many health facilities in Ethiopia lack the necessary infrastructure, such as reliable electricity. Although some structural data quality problems can be expected to improve with the increasing use of information technology, other issues may remain [8–10].

Health data are often expressed as prevalence or coverage and depend both on valid numerator information and on appropriate definition and assessment of the denominator. Data from the latest Ethiopian census in 2007 frequently serves as a basis for population estimates based on specific algorithms for each region [11,12, Ministry of Health, Ethiopia, personal communication, 2019].

Extensive routine data are collected in Ethiopia from all health-care levels and outside the health-care system [13]. The routine HMIS, sometimes referred to as the Routine Health Information System (RHIS), includes any regular data collection conducted in the health system and community with an interval of less than 1 year [14]. In the routine HMIS in Ethiopia, over 1000 data elements are reported monthly and around 400 quarterly. Further data are included on specific diseases such as HIV, and on activities such as quality assurance. Some data elements are compiled into indicators for the routine HMIS at the health centre level and above [11]. In 2017, the Ministry of Health (MOH) increased the number of routine HMIS indicators from 122 to 131 [11] (Supplementary Table S1). Information from the lower levels is also forwarded to the Ethiopian Public Health Institute in the Public Health Emergency Management framework, including the Maternal and Perinatal Death Surveillance and Response, and in the supply and procurement systems to the Ethiopian Pharmaceutical Supply Agency (Supplementary Figure S1). Vital events, such as births and deaths, are reported to the Vital Events Registration Authority that forwards data to the Central Statistical Agency.

Here we report an assessment of the quality of Ethiopian routine HMIS data. We compared 19 indicator definitions with the EDHS definitions, assessed completeness, internal consistency of national routine HMIS data, and external consistency with data from other sources, mainly the EDHS; and identified strengths, challenges, and opportunities for improvements in the routine HMIS data in Ethiopia. The rationale for our study was the pivotal role that can be played by a well-functioning routine HMIS for allocating resources and planning health care.

## Methods

### Study area, study population, and selected indicators

This study targeted national and regional routine HMIS data from Ethiopia. Ethiopia is the second-most populous country in Africa, with an estimated population of 110 million [15,16]. After re-structuring in 2020, there are 10 administrative regions as well as the two city administrations of Addis Ababa and Dire Dawa. Regions are sub-divided into 98 zones and further into 923 districts, *woreda*, which in turn are divided into the lowest administrative unit, *kebele*, having around 5000 inhabitants [17]. The MOH governs the health system with decentralized power to Regional Health Bureaux, which are responsible for management, coordination, and distribution of technical support to the lower levels. The health system has three levels (tiers): primary level (health posts, health centres, primary hospitals); secondary level (general hospitals); and third-level health care (specialised hospitals) [4]. Data flow starts at the point of service delivery, and data are compiled at the district, zonal, and regional offices before reaching the national level (Supplementary Figure S1).

Thirty-eight analysts from the MOH, the Ethiopian Public Health Institute, and the Ethiopian Pharmaceutical Supply Agency were selected for a two-year on-the-job capacity-development intervention, the Operational Research and Coaching for Analysts (ORCA) project. The ORCA-project was initiated by the MOH and implemented by the London School of Hygiene & Tropical Medicine. Workshops, training, and facilitated analytical work took place in parallel to the participants’ professional responsibilities. The work was performed in six thematic groups: Maternal Health, Neonatal Survival, Immunization, Child Nutrition, Malaria, and Tuberculosis. The ORCA thematic groups analysed 19 indicators and data elements that contribute to the indicators. Data sources used are shown in Table 1.

**Table 1.** Thematic groups, selected indicators and data elements for analysis and source documents used in this study

Thematic group	Indicator or data element analysed	Source documents for analysis of indicator or data element
Maternal Health	First antenatal care visit Four antenatal care visits Skilled birth attendance Postnatal care	HMIS 2014–2018^b^ EDHS 2016
Neonatal Survival	Early neonatal death at community Early institutional neonatal death rate Total number of births in the same *kebele*	HMIS 2014–2018^a^; EDHS 2016; Mini-EDHS 2019
Immunization	Pentavalent vaccine third dose Measles Fully immunized	HMIS 2014–2018^a^; EDHS 2016; HCMIS 2014–2018^a^
Child Nutrition	Vitamin A supplementation Deworming Severe acute malnutrition Growth monitoring promotion	HMIS 2012–2016^b^; EDHS 2016
Malaria	Suspected malaria Positive malaria All malaria	HMIS 2014–2018^a^; HCMIS 2014–2018^a^; World Malaria Report (WHO) 2015–2018
Tuberculosis	New and relapse tuberculosis Treated tuberculosis	HMIS 2014–2018^a^; HCMIS 2014–2018^a^

*^a^2014–2018 is Gregorian calendar 8 July 2014 to 7 July 2018 = Ethiopian Fiscal Year 2007–2010*

*^b^2012–2016 is Gregorian calendar 8 July 2012 to 7 July 2016 = Ethiopian Fiscal Year 2005–2008*

*EDHS = Ethiopian Demographic and Health Survey*

*HCMIS = Health Commodity Management Information System*

*HMIS = Health Management Information System*

*Kebele = the lowest administrative unit in Ethiopia, around 5000 persons*

*Penta = vaccine against Diphtheria, Tetanus, Pertussis, Hepatitis B, and Haemophilus Influenzae*

*WHO = World Health Organization*

### Data collection and analysis

The routine HMIS data from the national level, the nine regions that existed at the time of the study, and from the two city administrations were available from the MOH. Each thematic group identified appropriate source documents for external comparisons, such as the EDHS [1,2] (Table 1). The EDHS 2016 data on neonatal deaths were disaggregated to show early neonatal deaths by age in days. We also used data from the Health Commodities Management Information System (HCMIS) [4]. Data on pharmaceutical drugs and vaccines were expressed as standard person doses. For malaria, we compared the routine HMIS data with the World Health Organization (WHO) annual World Malaria Reports for 2015–2018 [18].

We used the second module of the WHO *Data Quality Review: a toolkit for facility data quality assessment*, and Excel sheets for data analysis, which were prepared using the same operational definitions as the WHO toolkit, see below [6,19]. The Ethiopian calendar differs from the Gregorian calendar by 7–8 years, and these differences, were considered in all comparisons (Table 1).

### Study design and operational definitions

The WHO toolkit [19] provides a method for analysing routine HMIS data using four dimensions of data quality. The first dimension concerns whether data are available (completeness, timeliness). The second looks at the internal consistency of routine HMIS data compared over time and between indicators that could be expected to have a relation, such as the number of women coming for antenatal care visits, deliveries and newborn vaccinations. The third dimension concerns the external consistency when routine HMIS data are compared with data from other sources such as the number of vaccinations compared to the supply of vaccines, or the ratio of routine HMIS performance over coverage in population surveys. The fourth dimension compares population estimates. In this study, we assessed the data quality dimensions of completeness, internal consistency, and external consistency and used population estimates to predict births.

*Definitions of indicators*. The definition of the indicators was analysed using the HMIS Indicators Reference Guide [11], and comparison with the indicators used by the EDHS [1].

*Data Quality Review dimension 1. Completeness of data*. We analysed the completeness of routine HMIS data for the 12 months of 1 year for the 14 indicators of all thematic groups, except malaria and tuberculosis. Completeness of data elements was defined as the presence of the reported aggregated data for the specified month.

*Data Quality Review dimension 2. Internal consistency of reported data (presence of outliers, consistency over time, and consistency between related indicators)*. A value within 2 to 3 standard deviations from the mean for the indicator over 12 months was considered a moderate outlier. A value of 3 or more standard deviations from the mean was considered an extreme outlier. The index year’s performance was divided by the average of the preceding 3 years to represent the consistency over time for each selected routine HMIS indicator or data element. The quality range was set at ±33%. Consequently, we defined consistency over time as a ratio from 0.66 to 1.33 and refer to these values as an acceptable value, ‘within the quality range.’ Using the WHO toolkit and based on the participants’ assumptions for each indicator, we considered whether the trend for each indicator was expected to be consistent, decreasing, or increasing. The indicators in the routine HMIS that were expected to have a logical relationship, such as fourth antenatal care visit and skilled birth attendance, or measles vaccinations and fully vaccinated, were used to evaluate the consistency between related indicators. The quality range for consistency between related indicators was set at ±10%.

*Data Quality Review dimension 3. External consistency of reported data*. The routine HMIS indicators or data elements were compared with data from other relevant data sources, mainly the EDHS [1,2]. The quality range for external consistency was set at ±33%.

## Results

### Definitions and alignment of indicators and data elements used

We compared definitions and reporting periods for the 19 routine indicators and data elements between the routine HMIS and the EDHS (Table 2). The four maternal health indicators were well aligned with the corresponding indicators in the latest EDHS. However, the routine HMIS defined a skilled birth attendant as a facility delivery with a skilled attendant who is a nurse, midwife, health officer, or doctor trained in deliveries, but not a health extension worker. In contrast, the EDHS 2016 categorised the birth attendants including the health extension worker, while the place of delivery was described in a separate indicator [1,11].

**Table 2.** Definitions of indicators and data elements in the routine Health Management Information System (HMIS) and the corresponding indicators in the Ethiopian Demographic and Health Survey (EDHS) 2016. HMIS indicator definitions from the Ministry of Health HMIS Indicators Reference Guide

Indicator used in this study	Definition of the numerator in the HMIS	Definition of the denominator in HMIS	Corresponding indicator in the EDHS 2016 *All survey data in relation to relevant population estimates*
Antenatal care 1	Number of pregnant women who received antenatal care first visit during the current pregnancy	Total number of expected pregnancies	First antenatal care visit
Antenatal care 4	Number of pregnant women that received four or more antenatal care visits	Total number of expected pregnancies	Four antenatal care visits
Skilled birth attendance	Number of births attended by skilled^a^ health personnel at a health facility^a^	Total number of expected deliveries	Proportion of women with deliveries five years prior to survey receiving assistance^a^ during delivery Place of delivery^a^ is a separate indicator
Postnatal care	Number of women who received postnatal care at least once within two days of delivery	Total number of expected deliveries	Proportion of women with deliveries two years prior to survey receiving postnatal care within two days
Early neonatal death at community	Number of deaths in the first week (day 0–6) of life^b^	Total number of live births^b^ in the same kebele	Neonatal mortality rate: the number of deaths in the first month of life^b^ regardless of place of death, for the five-year period preceding the survey
Early institutional neonatal death rate	Number of institutional neonatal deaths in the first week (day 0–6)^b^ of life	Total number of live births attended by skilled health attendants at health centers, clinics and hospitals	Not applicable (see neonatal mortality rate above)
Total births in the *kebele*^b^	Not applicable	The data element *Total number of births in the same kebele*^b^ is the denominator in the indicator *Early neonatal death in community* above	Fertility rate for the 3-year period preceding the study.
Penta 3^c^	Number of children below one year of age who have received the third dose of pentavalent vaccine	Estimated number of surviving^c^ infants	Coverage of third does of pentavalent vaccine “by appropriate age” (12 months)
Measles	Number of children under one year of age who have received the first dose of measles vaccine	Estimated number of surviving^c^ infants	Coverage of measles vaccination “by appropriate age” (12 months)
Fully immunized	Number of children who have received all routine vaccinations^c^ before their first birthday	Estimated number of surviving^c^ infants	Coverage of fully vaccinated children “all basic vaccinations”^c^ “by appropriate age”^c^
Vitamin A Supplementation	Total number of children aged 6–59 months who received two doses of vitamin A supplementation	Estimated number of children aged 6–59 months	Proportion of children 6–59 months who received Vitamin A once in the preceding 6 months
Deworming	Total number of children aged 24–59 months dewormed twice per year	Estimated number of children aged 24–59 months	Proportion of children 6^d −^59 months who received deworming once in the preceding six months
Severe acute malnutrition	Coverage^d^ of screening for severe acute malnutrition using mid-upper arm circumference or weight for height and number of children below five years of age classified to have severe acute malnutrition	Total number of children 0–60 months	Proportion of children with weight for height <-3 SD in the survey
Growth monitoring promotion	Number of children under two years of age weighed during growth monitoring promotion session	Estimated children under two years	None
Suspected malaria	Not an indicator in HMIS^e^	Not an indicator in HMIS^e^	None^e^
Positive malaria	The data element *Number of slides or RDTs positive for malaria* is the numerator in the indicator *Malaria positivity rate*	Not applicable	None^e^
All malaria	The data element *All malaria* is the numerator in the indicator *Morbidity attributed to malaria*	Not applicable^e^	None^e^
New and relapse tuberculosis^f^	The data element *Number of new and relapse cases* is the numerator in the indicator *Tuberculosis case detection rate*	Not applicable^f^	None^f^
Treated tuberculosis^f^	Not applicable^f^	The data element *Treated tuberculosis cases* is the denominator of the indicator *Treatment success of TB patients who received community-based treatment suppor*t	None^f^

*EDHS = Ethiopian Demographic and Health Survey*

*HMIS = Health Management Information System*

*Penta = vaccine against Diphtheria, Tetanus, Pertussis, Hepatitis B, and Haemophilus Influenzae*

*^a^In the HMIS, a skilled attendant is defined as a health professional (such as a midwife, nurse, health officer or doctor) who has been trained in the skills needed to manage normal (uncomplicated) pregnancies, childbirth and the immediate postnatal period. HMIS also requires that the delivery takes place at a facility. HMIS does not include health extension workers (HEW) or traditional birth attendants (TBA) among skilled attendants. In the EDHS, a skilled attendant is defined as a doctor, nurse, midwife, health officer, or HEW. It does not include TBA.*

*^b^Early neonatal death at community (ECND) and early institutional neonatal death rate (EIND) are reported separately so that ECND and EIND together make up the total early neonatal death. There is no indicator for deaths in day 0–28 in HMIS. In the EDHS, the deaths within day 0–6 can be disaggregated from the total neonatal deaths in day 0–28. Births are registered in another system, the Vital events registration, by the local administration in the kebele (lowest administrative level) that collects data on births and deaths from health facilities and community. The total number of births in the same kebele is the denominator of ECND.*

*^c^HMIS considers a child as having full immunization coverage if he or she received the following vaccines that are in the current Expanded Program of Immunization in Ethiopia: BCG (tuberculosis), 3 doses of Penta, 3 doses of oral polio-vaccine, 3 doses of PCV-vaccine (pneumococcal conjugate), 2 doses of rota-vaccine, 1 dose of IPV-vaccine (inactivated polio vaccine) and 1 dose of measles-vaccine before the age of 1 year. In the EDHS, ‘all basic vaccinations’ is defined as one dose of BCG, three doses of Penta, three doses of polio vaccine and one dose of measles vaccine, whereas ‘all age-appropriate vaccinations’ also include the newer vaccinations: 3 doses of PCV and 2 doses of rota, but not IPV. The denominator ‘estimated number of surviving infants’ refers to the population estimate and is used for denominators as stated in the table (MOH, personal communication). In contrast, the guideline states that the denominator ‘total number of surviving infants’, meaning infants who survive to their first birthday, to be used for measles and fully immunized, but not for Penta 3 where the estimated number of infants is in the guideline.*

*^d^In EDHS, the reported deworming among children 6–24 months for the younger ages may represent treatments rather than prophylaxis. In HMIS, the indicator severe acute malnutrition (SAM) is the proportion of children screened for SAM (coverage) and findings are further classified into prevalence of SAM, using the definitions Middle Upper Arm Circumference (MUAC) <11 cm or weight for height <70% of the median, or <-3 Z score (used in health centers and hospitals) and/or bilateral pitting edema (used in all health facilities). The growth monitoring promotion (GMP) is a preventive activity that includes measuring, analyzing and counseling on nutrition and is therefore not the same as the SAM-screening.*

*^e^The ORCA malaria group studied the suspected, confirmed, and all malaria (=”total malaria”) as reported in the HMIS-framework. These measurements are data elements and ‘All malaria’ is the numerator of the corresponding burden-of-disease HMIS-indicator ‘Morbidity attributed to malaria’, and the estimated total population is the denominator. ‘All malaria’ is ‘positive malaria’ with the addition of ‘Clinical diagnosis of malaria’ (=presumed treatment) (Supplementary Figure S1). In the EDHS, only the use of anti-malarial drugs is surveyed.*

*^f^The ORCA tuberculosis group studied the new and relapse cases and treated cases of tuberculosis as reported in the HMIS (Supplementary Figure S2). Number of new and relapse cases is the numerator of ‘Tuberculosis case detection rate’ where the annual WHO-estimate is the denominator. The treated tuberculosis is the denominator of the indicator ‘Treatment success of TB patients who received community-based treatment’ which aims at determining the proportion of all forms of new TB cases successfully treated (cured plus completed treatment) among those who received treatment adherence support at community for at least full course of the continuation phase treatment. Tuberculosis is not investigated in the EDHS.*

The definition of indicators regarding neonatal mortality were poorly aligned. The routine HMIS reported neonatal deaths as deaths on days 0–6 after birth, but did not include deaths from day 7–28. The data element ‘total number of births in the same kebele’ was used as the denominator to determine the community’s early neonatal death rate. The total number of births in the same kebele included all births, whether institutional or at home [11]. In the EDHS, a neonatal death was defined as a death occurring on day 0–28 [1].

The definition of fully vaccinated children was similar in the routine HMIS and the EDHS at the time of the comparisons. Since then, some new vaccines have been added to the HMIS definition of fully vaccinated (Table 2). The EDHS presented figures of all basic vaccinations assessed in the group 12–23 months, and ‘vaccinated by appropriate age,’ which can be compared to the routine HMIS data for fully vaccinated children <1 year. The denominators used in the routine HMIS for coverage of the third pentavalent vaccination, measles vaccine, and ‘fully vaccinated’ were the estimated number of surviving infants for all indicators (Ministry of Health, Ethiopia, 2020, personal communication). This algorithm-based estimate was the practice despite guidelines that stated the total number of surviving infants should be used for measles vaccine and ‘fully vaccinated’, implying actual children who survived their first birthday. The HMIS defined Vitamin A supplementation as two doses in 1 year, whereas the EDHS reported data on one dose of Vitamin A in 6 months. Also, there were minor differences in age groups. The HMIS tuberculosis indicators were numerous [16] and complex, with both numerators and denominators being both highly specific and complex, such as ‘latent tuberculosis infection treatment coverage for under 5-years children who are contacts of pulmonary TB cases’ (Supplementary Table S1). For malaria and tuberculosis, there were no indicators in the EDHS.

### Data quality review dimension 1: completeness of data

Data on immunization in 2017/18 were complete, as were data on child nutrition indicators for 2015/16. Data on skilled birth attendance and first and fourth antenatal care visits 2017/18 were complete, but postnatal care showed 2 months with missing data, both from the same region (Table 3(a)). Data on early neonatal deaths in the community for 2014/2015 were not reported from one city administration (Table 3(b)).

**Table 3a.** Total number of women coming for postnatal care within two days of birth by region and month in the routine Health Management Information System, July 2017-June 2018. Internal consistency. Outliers within 2–3 standard definitions from the mean value per region or city administration are underlined and extreme outlier >3 standard deviation from the mean shown in bold

	Month											
Area	1	2	3	4	5	6	7	8	9	10	11	12
Addis Ababa (city)	7931	8593	7968	7831	6634	7885	7574	7751	8200	8067	10,476	9411
Afar region	2035	2166	2257	2412	2352	2366	2090	2417	2122	2220	1986	2025
Amhara region	41,038	46,691	43,515	43,274	45,177	46,155	47,399	46,338	45,133	44,393	49,375	40,134
Benishangul-Gumuz region	2658	2584	2737	2713	2591	3040	3069	2185	2190	2426	2235	1886
Dire Dawa (city)	765	854	847	819	664	1887	858	969	1153	833	887	816
Gambela region	376	339	493	453	440	378	363	412	330	301	448	408
Harari region	371	368	367	417	511	420	450	664	352	463	598	526
Oromia region	86,731	91,638	87,213	86,251	81,345	82,934	73,409	81,902	82,561	78,682	79,480	80,085
Somali region	8725	7388	7158	7225	6769	9793	9780	9005	9350	5511	6191	5518
SNNP region	44,133	48,510	-	-	**0**	48,117	48,501	50,728	50,710	52,918	54,677	50,198
Tigray region	9532	10,455	10,853	10,928	10,357	11,849	11,581	10,884	11,205	11,050	11,644	10,377

*- No value*

*SNNP = Southern Nations, Nationalities, and Peoples*

**Table 3b.** Total number of early neonatal death at community by region and month in the routine Health Management Information System, July 2014-June 2015. Internal consistency. Outliers within 2–3 standard definitions from the mean value per region or city administration are underlined

	Month											
Area	1	2	3	4	5	6	7	8	9	10	11	12
Addis Ababa (city)	-	-	-	-	-	-	-	-	-	-	-	-
Afar region	2	0	0	2	5	2	4	3	4	0	1	0
Amhara region	8	4	34	19	10	13	14	17	10	13	8	42
Benishangul-Gumuz region	3	1	16	6	5	4	5	6	20	1	11	25
Dire Dawa (city)	0	0	0	0	0	0	0	0	0	0	0	0
Gambela region	0	0	0	6	0	1	0	0	0	1	0	3
Harari region	0	0	0	0	0	0	0	0	0	0	0	1
Oromia region	58	43	44	82	22	59	28	21	14	28	16	24
Somali region	-	-	-	0	-	-	0	0	0	1	0	0
SNNP region	1041	43	1701	2008	20	9	160	13	17	24	145	4
Tigray region	19	5	8	3	5	8	7	4	7	5	5	2

*- No value*

*SNNP = Southern Nations, Nationalities, and Peoples*

### Data quality review dimension 2: internal consistency

#### Presence of outliers

Data on postnatal care showed a few outliers (Table 3(a)), including one extreme outlier with a recorded value of zero. Data on births (data not shown) and early neonatal deaths in the community (Table 3(b)) were both prone to outliers. For Vitamin A supplementation, there were both extreme and moderate outliers, and outliers were also present for deworming. Outliers were often seen in month six and month 12, corresponding to reporting periods.

#### Consistency over time

**Figure 1.**
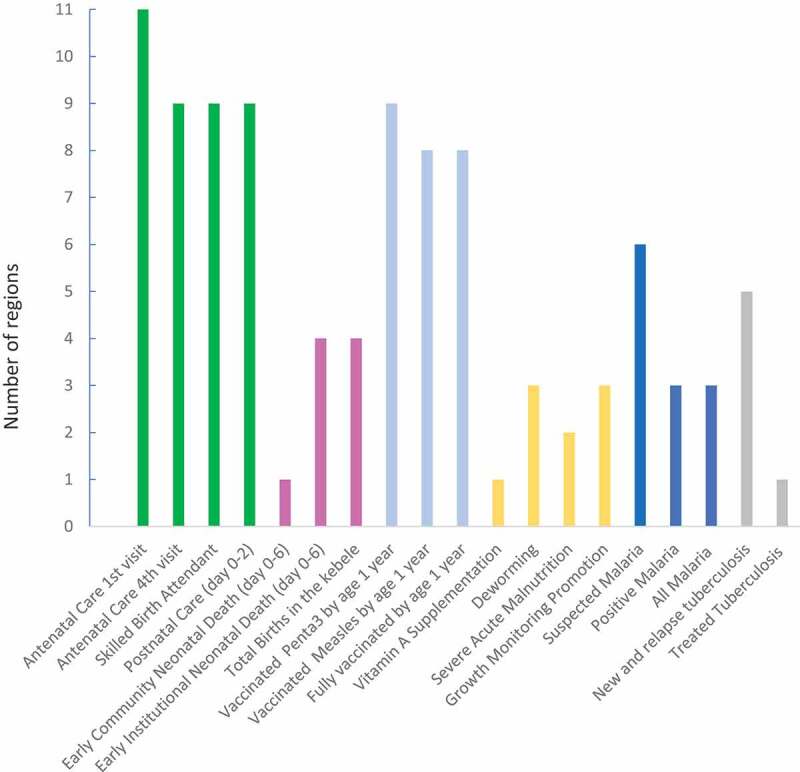
Number of regions and city administrations (*n* = 11) with internal consistency over time per routine Health Management Information System indicator or data element (*n* = 19)

The routine HMIS indicator data for 1 year were compared to the average of the preceding 3 years for all 19 indicators (Supplementary Table S2). There was consistency over time for maternal health and immunization in most regions and city administrations. In contrast, neonatal health and child nutrition indicators showed consistency over time in less than half of the regions or city administrations (Figure 1). All regions but one, showed an expected positive trend in maternal health indicators. However, all remained within the quality range of 33% of the average of the three preceding years (Supplementary Table S2).

Kebele = lowest administrative level

**Figure 2.**
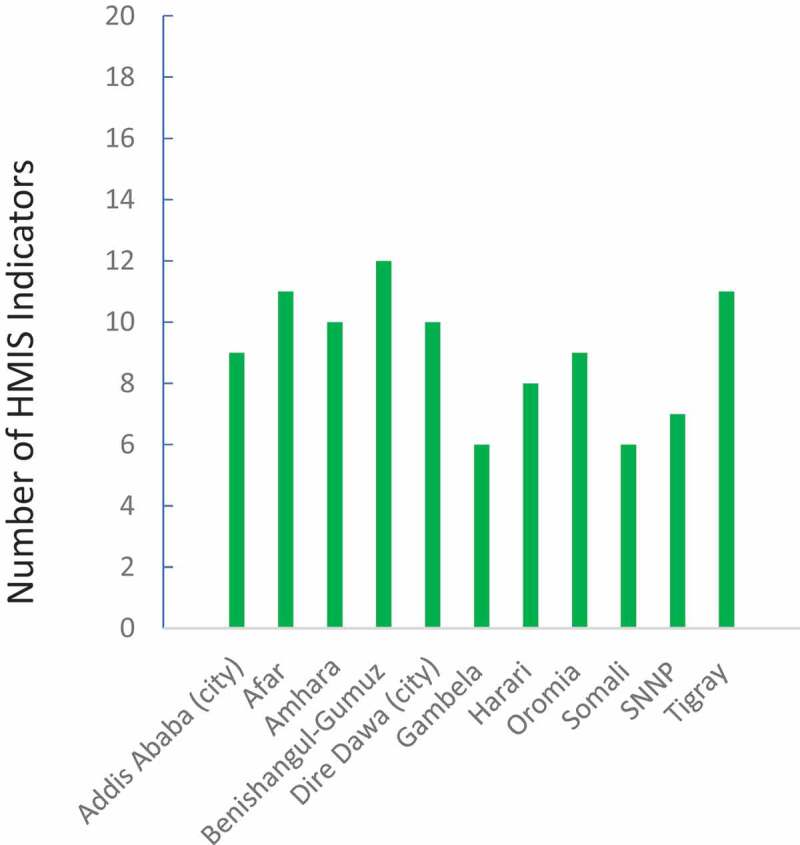
Number of routine Health Management Information System indicators (*n* = 19) that showed internal consistency over time per region or city administration (*n* = 11)

No region or city administration showed consistency over time for all selected indicators. The consistency over time ranged from six to 12 out of the 19 indicators (Figure 2).

SNNP = Southern Nations, Nationalities, and Peoples

#### Consistency between related indicators

There was a particular inconsistency in denominators: only 0.7 million births in the same kebele were recorded, yet 2.4 million early postnatal care visits and 2.7 million children vaccinated with the third dose of pentavalent vaccine were reported in 2017/2018. The internal consistency between children vaccinated with the third dose of pentavalent vaccine and measles was in the quality range in all regions. The number of treated tuberculosis cases was higher than the number of new and relapse cases in two regions, and the reverse was seen in one region (data not shown).

### Data quality review dimension 3: external consistency

**Figure 3.**
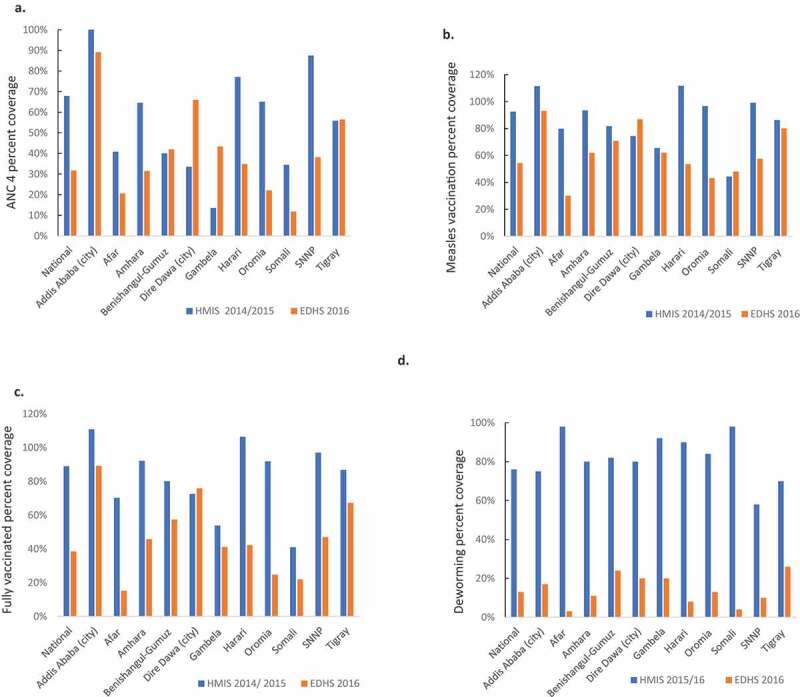
External consistency comparing the routine Health Management Information System (HMIS) data 2014/15 or 2015/16 (Ethiopian fiscal year 2007 or 2008) with the Ethiopian Demographic and Health Survey (EDHS) 2016 for (a) coverage of four antenatal care visits, (b) coverage of vaccinations against measles, (c) fully vaccinated 1-year old children and, (d) coverage of deworming of children

The number of women attending four antenatal care visits varied in a non-systematic way. Nevertheless, most regions reported higher numbers in the routine HMIS than was recorded in the EDHS (Figure 3(a)). Similar patterns were noted for the first antenatal care visit and early postnatal care (data not shown). The combined national institutional and community early neonatal deaths in the routine HMIS were 11 755 children in 2014/15, 7 591 children in 2015/16, and 8 117 children in 2016/17. The neonatal mortality rate of 30 per 1000 live births [2], population 109 million, and crude birth rate of 32.2/1000 from 2018 [15] would result in an expected number of 3.5 million annual births and 100 000 deaths within the first month of life every year. With 75% of neonatal deaths occurring in the first week [1], the expected number of annual early neonatal deaths on day 0–6 of life would be around 75 000. Hence, our results indicate that only one in eight early neonatal deaths were reported in the routine HMIS.

The immunization indicators generally showed good agreement when comparing the routine HMIS with the Ethiopian Demographic and Health Survey (Figure 3(b)). Nevertheless, this external consistency was lower for the indicator ‘fully immunized children’ (Figure 3(c)) and for ‘vaccinated with the third dose of the pentavalent vaccine’ and for the number of vaccine doses distributed (Supplementary Figure S2). The child nutrition indicators, such as deworming, showed weak consistency when comparing the routine HMIS with the Ethiopian Demographic and Health Survey 2016 (Figure 3(d)). Indicators suggested a decreasing incidence of malaria over time, but the number of prescribed antimalarials was higher than malaria cases reported in the routine HMIS (data not shown).

### Data quality review dimension 4: external comparison of population data

The 0.7 million births reported in 2017/2018 routine HMIS was compared to the crude birth rate of 32.6 per 1000 population [15]. With Ethiopia´s population of 110 million [15,16], the expected number of births was 3.6 million per year, and hence we estimate only 19% of births were reported in the routine HMIS.

## Discussion

This study of routine HMIS data quality in Ethiopia showed quality problems for all indicators, especially compared to external information sources. Indicators regarding some aspects of maternal health care and immunization were mostly complete and consistent over time. Indicators on child nutrition, malaria, and tuberculosis were more prone to outliers, less consistent over time, and showed major differences when triangulated with other information sources. Most notably, the indicators on births and neonatal mortality were incomplete and had very low internal and external consistency. We also identified regional differences in the quality of the routine HMIS data.

### Indicator definitions and reporting guidelines

The indicators and data elements reflect an ambition to improve health care and a desire to capture new interventions. However, including a large number of complex indicators and data elements may also contribute to the burden of reporting and the risk of errors. The HMIS Indicators Reference Guide for routine HMIS [11] provides information in English on the definition of indicators. Work is ongoing efforts to translate the national guidelines to the major languages used in Ethiopia, and this is likely to increase the understanding of indicator definitions at the health system’s lower levels.

Several issues on reporting may need to be addressed: double-reporting, if the first visit by a pregnant woman for antenatal care was registered at a health post and again at a health centre, or over-reporting, if an antenatal care visit close to the expected date of delivery is recorded as the fourth visit irrespective of the number of visits. Another example of a reporting issue is vitamin A supplementation. There may be a lack of clarity over whether the number reported represents the number of children or the number of doses of Vitamin A provided. The data element ‘total number of births in the same kebele’ includes both institutional births and births in the community: this data element was severely under-reported. The reasons for not reporting neonatal deaths in the routine HMIS need to be further explored as part of the efforts to reach the global target of less than 12 deaths per 1000 live births by 2030 [20–22].

The WHO toolkit provided a useful method for analysing data. Triangulating routine HMIS data can increase the awareness of quality problems and help go beyond analysing the accuracy of reporting within the routine HMIS [23,24]. To some extent, discrepancies in external consistency may be due to reporting errors that affect indicators to varying degrees in the surveys used for comparison. A woman likely remembers where she last gave birth, but the exact number of antenatal visits may be more challenging to capture [25]. Integrated and continuous surveying by permanent survey teams has been suggested as a model for countries aiming at improving their routine HMIS [26]. An initial step could be to use the already existing surveys for these comparisons, despite the long time interval between surveys which would hamper the analyses. One recent scoping review on child immunization in Ethiopia identified considerable discrepancies in reports from various sources, and used this information to identify research priorities, consulting an expert panel of stakeholders [27].

Some strengths of this study are that we used a WHO data quality review toolkit [18] and that the work was led by analysts familiar with the routine HMIS data. Implementation research is likely more efficient when conducted by the usual implementation agencies, as in this study [28]. We compared results for indicators defined differently by different but compatible sources, and we explored these differences in indicator definitions. Timeliness is one aspect of data quality that was not addressed in this study. In our research, the newly introduced vital events registration was not assessed. Also, we did not evaluate data quality at the district and facility levels.

## Conclusion

We analysed the consistency of routine HMIS data and identified strengths and challenges in Ethiopia’s routine HMIS data. We conclude that the internal consistency varied between indicators and regions. In general, internal consistency in the routine HMIS was better for indicators on maternal health and immunization than for other indicators. Internal consistency was better than external consistency, where routine HMIS data were compared with data from other sources, mostly survey data. The lack of external consistency suggests quality problems in the routine HMIS data that go beyond correct reporting. We also conclude that the uncertainty of population estimates makes a major contribution to discrepancies. We suggest future reality-checks with triangulation of routine HMIS and data from other sources and alignment of routine HMIS indicators with those of the EDHS to increase comparability. Together with the ongoing digitalisation that is part of the Information Revolution brought forward by the MOH, our suggestions may improve the routine HMIS data quality. In Ethiopia and globally, improved routine HMIS data are pivotal to achieving universal health coverage [29].

## Supplementary Material

Supplemental Material

## Paper context

Continuous data generated at the point of service delivery is pivotal for decision-making to ensure quality, equality, and accountability in health services. We analysed routine Health Management Information System data and compared it with surveys. We identified variations in quality between indicators and regions and issues on the definitions and complexity of indicators. Data on coverage were affected by the low quality of population estimates. We provide recommendations to strengthen the routine Health Management Information System.
