# Supplementary material for: Routine health management information system data in Ethiopia: consistency, trends, and challenges
Source: Glob Health Action. 2021 Jan 15;14(1):1868961. doi: 10.1080/16549716.2020.1868961 (PMC7833046; doi:10.1080/16549716.2020.1868961)
Supplement: Supplemental Material [file ZGHA_A_1868961_SM9413.zip › Supplementary/Supplementary Figure 1.docx]

**Figure 1: Number of regions and city administrations (n=11) with internal consistency over time per routine Health Management Information System indicator or data element (n=19)**

*Kebele=lowest administrative level*
