# Supplementary material for: Routine health management information system data in Ethiopia: consistency, trends, and challenges
Source: Glob Health Action. 2021 Jan 15;14(1):1868961. doi: 10.1080/16549716.2020.1868961 (PMC7833046; doi:10.1080/16549716.2020.1868961)
Supplement: Supplemental Material [file ZGHA_A_1868961_SM9413.zip › Supplementary/Supplementary Figure 2.docx]

Number of HMIS Indicators

**Figure 2: Number of routine Health Management Information System indicators (n=19) that showed internal consistency over time per region or city administration (n=11)**

*SNNP= Southern Nations, Nationalities, and Peoples*
