# Supplementary material for: Routine health management information system data in Ethiopia: consistency, trends, and challenges
Source: Glob Health Action. 2021 Jan 15;14(1):1868961. doi: 10.1080/16549716.2020.1868961 (PMC7833046; doi:10.1080/16549716.2020.1868961)
Supplement: Supplemental Material [file ZGHA_A_1868961_SM9413.zip › Supplementary/Supplementary Table 1.docx]

**Table 1. Thematic groups, selected indicators and data elements for analysis and source documents used in this study**

| **Thematic group** | **Indicator or data element analysed** | **Source documents for analysis of indicator or data element** |
| --- | --- | --- |
| Maternal Health | First antenatal care visit  Four antenatal care visits  Skilled birth attendance  Postnatal care | HMIS 2014-2018^[[1]](#footnote-1)^  EDHS 2016 |
| Neonatal Survival | Early neonatal death at community  Early institutional neonatal death rate  Total number of births in the same *kebele* | HMIS 2014-2018^1^;  EDHS 2016;  Mini-EDHS 2019 |
| Immunization | Pentavalent vaccine third dose  Measles  Fully immunized | HMIS 2014-2018^1^;  EDHS 2016;  HCMIS 2014-2018^1^ |
| Child Nutrition | Vitamin A supplementation  Deworming  Severe acute malnutrition  Growth monitoring promotion | HMIS 2012–2016^2^;  EDHS 2016 |
| Malaria | Suspected malaria  Positive malaria  All malaria | HMIS 2014-2018^1^; HCMIS 2014-2018^1^; World Malaria Report (WHO) 2015-2018 |
| Tuberculosis | New and relapse tuberculosis  Treated tuberculosis | HMIS 2014-2018^1^; HCMIS  2014-2018^1^ |

*^1^2014-2018 is Gregorian calendar July 8, 2014 to July 7, 2018=Ethiopian Fiscal Year 2007-2010*

*^2^2012-2016 is Gregorian calendar July 8, 2012 to July 7, 2016=Ethiopian Fiscal Year 2005-2008*

*EDHS= Ethiopian Demographic and Health Survey*

*HCMIS = Health Commodity Management Information System*

*HMIS = Health Management Information System*

*Kebele= the lowest administrative unit in Ethiopia, around 5000 persons*

*Penta = vaccine against Diphtheria, Tetanus, Pertussis, Hepatitis B and Haemophilus Influenzae*

*WHO = World Health Organization*

1. [↑](#footnote-ref-1)
