# Supplementary material for: Routine health management information system data in Ethiopia: consistency, trends, and challenges
Source: Glob Health Action. 2021 Jan 15;14(1):1868961. doi: 10.1080/16549716.2020.1868961 (PMC7833046; doi:10.1080/16549716.2020.1868961)
Supplement: Supplemental Material [file ZGHA_A_1868961_SM9413.zip › Supplementary/Supplementary Table 2.docx]

**Table 2. Definitions of indicators and data elements in the routine Health Management Information System (HMIS) and the corresponding indicators in the Ethiopian Demographic and Health Survey (EDHS) 2016.**

HMIS indicator definitions from the Ministry of Health HMIS Indicators Reference Guide

| **Indicator used in this study** | **Definition of the numerator in the HMIS** | **Definition of the denominator in HMIS** | **Corresponding indicator in the EDHS 2016**  *All survey data in relation to relevant population estimates* |
| --- | --- | --- | --- |
| Antenatal care 1 | Number of pregnant women who received antenatal care first visit during the current pregnancy | Total number of expected pregnancies | First antenatal care visit |
| Antenatal care 4 | Number of pregnant women that received four or more antenatal care visits | Total number of expected pregnancies | Four antenatal care visits |
| Skilled birth attendance | Number of births attended by skilled^1^ health personnel  at a health facility^1^ | Total number of expected deliveries | Proportion of women with deliveries five years prior to survey receiving assistance^1^ during delivery  Place of delivery^1^ is a separate indicator |
| Postnatal care | Number of women who received postnatal care at least once within two days of delivery | Total number of expected deliveries | Proportion of women with deliveries two years prior to survey receiving postnatal care within two days |
| Early neonatal death at community | Number of deaths in the first week (day 0-6) of life^2^ | Total number of live births^2^ in the same kebele | Neonatal mortality rate: the number of deaths in the first month of life^2^ regardless of place of death, for the five-year period preceding the survey |
| Early institutional neonatal death rate | Number of institutional neonatal deaths in the first week (day 0-6)^2^ of life | Total number of live births attended by skilled health attendants at health centers, clinics and hospitals | Not applicable (see neonatal mortality rate above) |
| Total births in the *kebele*^2^ | Not applicable | The data element *Total number of births in the same kebele*^2^ is the denominator in the indicator *Early neonatal death in community* above | Fertility rate for the 3-year period preceding the study. |
| Penta 3^3^ | Number of children below one year of age who have received the third dose of pentavalent vaccine | Estimated number of surviving^3^ infants | Coverage of third does of pentavalent vaccine “by appropriate age” (12 months) |
| Measles | Number of children under one year of age who have received the first dose of measles vaccine | Estimated number of surviving^3^ infants | Coverage of measles vaccination “by appropriate age” (12 months) |
| Fully immunized | Number of children who have received all routine vaccinations^3^ before their first birthday | Estimated number of surviving^3^ infants | Coverage of fully vaccinated children “all basic vaccinations”^3^ “by appropriate age”^3^ |
| Vitamin A Supplementation | Total number of children aged 6-59 months who received two doses of vitamin A supplementation | Estimated number of children aged 6-59 months | Proportion of children 6-59 months who received Vitamin A once in the preceding 6 months |
| Deworming | Total number of children aged 24-59 months dewormed twice per year | Estimated number of children aged 24-59 months | Proportion of children 6^4^ -59 months who received deworming once in the preceding six months |
| Severe acute malnutrition | Coverage^4^ of screening for severe acute malnutrition using mid-upper arm circumference or weight for height and number of children below five years of age classified to have severe acute malnutrition | Total number of children 0-60 months | Proportion of children with weight for height <-3 SD in the survey |
| Growth monitoring promotion | Number of children under two years of age weighed during growth monitoring promotion session | Estimated children under two years | None |
| Suspected malaria | Not an indicator in HMIS^5^ | Not an indicator in HMIS^5^ | None^5^ |
| Positive malaria | The data element *Number of slides or RDTs positive for malaria* is the numerator in the indicator *Malaria positivity rate* | Not applicable | None^5^ |
| All malaria | The data element *All malaria* is the numerator in the indicator *Morbidity attributed to malaria* | Not applicable^5^ | None^5^ |
| New and relapse tuberculosis^6^ | The data element *Number of new and relapse cases* is the numerator in the indicator *Tuberculosis case detection rate* | Not applicable^6^ | None^6^ |
| Treated tuberculosis^6^ | Not applicable^6^ | The data element *Treated tuberculosis cases* is the denominator of the indicator *Treatment success of TB patients who received community-based treatment suppor*t | None^6^ |

*EDHS = Ethiopian Demographic and Health Survey*

*HMIS = Health Management Information System*

*Penta=vaccine against Diphtheria, Tetanus, Pertussis, Hepatitis B and Haemophilus Influenzae*

*^1^In the HMIS, a skilled attendant is defined as a health professional (such as a midwife, nurse, health officer or doctor) who has been trained in the skills needed to manage normal (uncomplicated) pregnancies, childbirth and the immediate postnatal period. HMIS also requires that the delivery takes place at a facility. HMIS does not include health extension workers (HEW) or traditional birth attendants (TBA) among skilled attendants. In the EDHS, a skilled attendant is defined as a doctor, nurse, midwife, health officer, or HEW. It does not include TBA.*

*^2^Early neonatal death at community (ECND) and early institutional neonatal death rate (EIND) are reported separately so that ECND and EIND together make up the total early neonatal death. There is no indicator for deaths in day 0-28 in HMIS. In the EDHS, the deaths within day 0-6 can be disaggregated from the total neonatal deaths in day 0-28. Births are registered in another system, the Vital events registration, by the local administration in the kebele (lowest administrative level) that collects data on births and deaths from health facilities and community. The total number of births in the same kebele is the denominator of ECND.*

*^3^HMIS considers a child as having full immunization coverage if he or she received the following vaccines that are in the current Expanded Program of Immunization in Ethiopia: BCG (tuberculosis), 3 doses of Penta, 3 doses of oral polio-vaccine, 3 doses of PCV-vaccine (pneumococcal conjugate), 2 doses of rota-vaccine, 1 dose of IPV-vaccine (inactivated polio vaccine) and 1 dose of measles-vaccine before the age of 1 year. In the EDHS, “all basic vaccinations” is defined as one dose of BCG, three doses of Penta, three doses of polio vaccine and one dose of measles vaccine, whereas “all age-appropriate vaccinations” also include the newer vaccinations: 3 doses of PCV and 2 doses of rota, but not IPV. The denominator “estimated number of surviving infants” refers to the population estimate and is used for denominators as stated in the table (MOH, personal communication). In contrast, the guideline states that the denominator “total number of surviving infants”, meaning infants who survive to their first birthday, to be used for measles and fully immunized, but not for Penta 3 where the estimated number of infants is in the guideline.*

*^4^ In EDHS, the reported deworming among children 6-24 months for the younger ages may represent treatments rather than prophylaxis. In HMIS, the indicator severe acute malnutrition (SAM) is the proportion of children screened for SAM (coverage) and findings are further classified into prevalence of SAM, using the definitions Middle Upper Arm Circumference (MUAC) <11cm or weight for height <70% of the median, or <-3 Z score (used in health centers and hospitals) and/or bilateral pitting edema (used in all health facilities). The growth monitoring promotion (GMP) is a preventive activity that includes measuring, analyzing and counseling on nutrition and is therefore not the same as the SAM-screening.*

*^5^The ORCA malaria group studied the suspected, confirmed, and all malaria (=”total malaria”) as reported in the HMIS-framework. These measurements are data elements and “All malaria” is the numerator of the corresponding burden-of-disease HMIS-indicator “Morbidity attributed to malaria”, and the estimated total population is the denominator. “All malaria” is “positive malaria” with the addition of “Clinical diagnosis of malaria” (=presumed treatment) (Supplementary Figure 1). In the EDHS, only the use of anti-malarial drugs is surveyed.*

*^6^ The ORCA tuberculosis group studied the new and relapse cases and treated cases of tuberculosis as reported in the HMIS (Supplementary Figure 2). Number of new and relapse cases is the numerator of “Tuberculosis case detection rate” where the annual WHO-estimate is the denominator. The treated tuberculosis is the denominator of the indicator “Treatment success of TB patients who received community-based treatment” which aims at determining the proportion of all forms of new TB cases successfully treated (cured plus completed treatment) among those who received treatment adherence support at community for at least full course of the continuation phase treatment. Tuberculosis is not investigated in the EDHS.*
